# Thoracic Aortic Injury: Embolization of the Tenth Intercostal Artery and Endovascular Treatment in a Young Woman after Posterior Spinal Instrumentation

**DOI:** 10.1155/2015/531201

**Published:** 2015-05-07

**Authors:** Konstantinos Lagios, Georgios Karaolanis, Theodossios Perdikides, Theodoros Bazinas, Nikolaos Kouris, Spiros Sfikas, Odysseas Paxinos

**Affiliations:** ^1^Interventional Radiology and Neuroradiology Department, 251 Hellenic Air Force Hospital, Athens, Greece; ^2^Vascular Surgery Department, 251 Hellenic Air Force Hospital, Athens, Greece; ^3^Anesthesiology Department, 251 Hellenic Air Force Hospital, Athens, Greece; ^4^Orthopaedic Department, 251 Hellenic Air Force Hospital, Athens, Greece

## Abstract

Iatrogenic aortic injuries are rare and well-recognized complications of a variety of procedures, including spinal surgery. The placement of pedicle screws is sometimes associated with devastating consequences. Aortic perforation with rapid hematoma formation and delayed aortic trauma leading to pseudoaneurysm formation have been described in the literature. A case describing a significant time interval between iatrogenic aortic injury and diagnosis in the absence of pseudoaneurysm formation is described in this paper and, according to our knowledge, is unique in the literature. The aortic injury was successfully treated, selecting the appropriate graft and, as a consequence, normal spinal cord blood flow was achieved.

## 1. Introduction

Iatrogenic aortic injuries are rare and well-recognized complications of a variety of procedures, including spinal surgery [1]. Vaccaro et al. have reported that placing pedicle screws in the thoracic spine exposes the patient to the risk of major vascular injury [2]. On the one hand, aortic perforation is frequently associated either with hemodynamic instability, due to acute hemorrhage, or with rapid hematoma formation, or both. On the other hand, delayed aortic trauma can evolve into pseudoaneurysm formation due to weakness in the aortic wall. Different invasive strategies, of which endovascular approaches with the use of stents are the dominant, are available for the protection from life-threatening bleeding due to the removal of pedicle screws [3]. This paper presents a case of uncommon thoracic aortic injury at the orifice of tenth (T10) intercostal artery, caused by a misplaced pedicle screw, which has been successfully treated using an endovascular procedure.

## 2. Case Presentation

A 22-year-old woman was admitted to our hospital complaining of acute thoracic pain. She had undergone a corrective spinal surgery six years ago because of idiopathic scoliosis. The surgical approach was performed through the posterior thoracolumbar spine. The instrumentation consisted of two rods fixed by pedicle screws from T10 to L4. The procedure was well tolerated and the patient had an uneventful postoperative period. She also mentioned an episode of acute abdominal pain four years ago treated in another hospital. Abdominal radiography and laboratory findings were then negative. She mentioned no other comorbidities.

**Figure 1:**
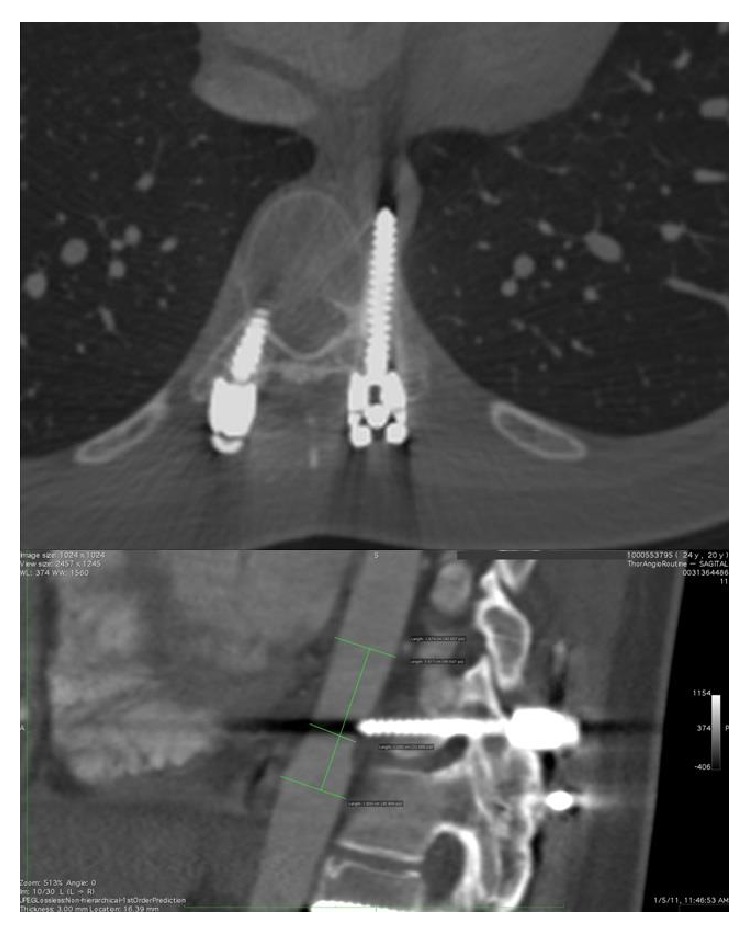
Preoperative CTA reveals the presence of the pedicle screw at the T10 level of the thoracic aorta.

On examination, she was hemodynamically stable. A contrast-enhanced computed tomography angiography (CTA) revealed a malpositioned pedicle screw and subsequently evaluated the status of the aortic tree (Figure 1). Moreover, digital angiography was performed to evaluate the correlation between the injured segment and the intercostal artery, as well as the perfusion state of the spinal cord. The pedicle screw impinged on the aortic wall at the orifice of the left T10 intercostal artery and close to a posterior radiculomedullary artery, whereas the Adamkiewicz artery with a posterior radiculomedullary artery originated from the right T9. Surgical options (endovascular, hybrid, or open procedures) were discussed with the patient. The endovascular technique was then decided and an endovascular suite was prepared, in case open surgical repair was needed.

## 3. Surgical Technique

**Figure 2:**
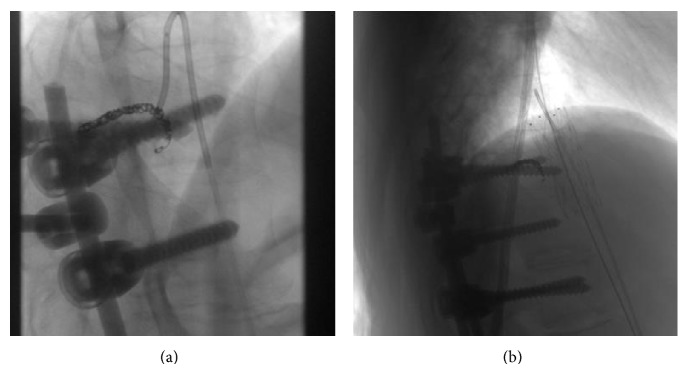
(a) Embolization of the T10 left intercostal artery with microcoils filling 3 cm of the vessel length, using the 4 fr Simmons catheter. (b) Placement of the stent graft by covering the site of penetration with the metal-free, central part of the stent.

**Figure 3:**
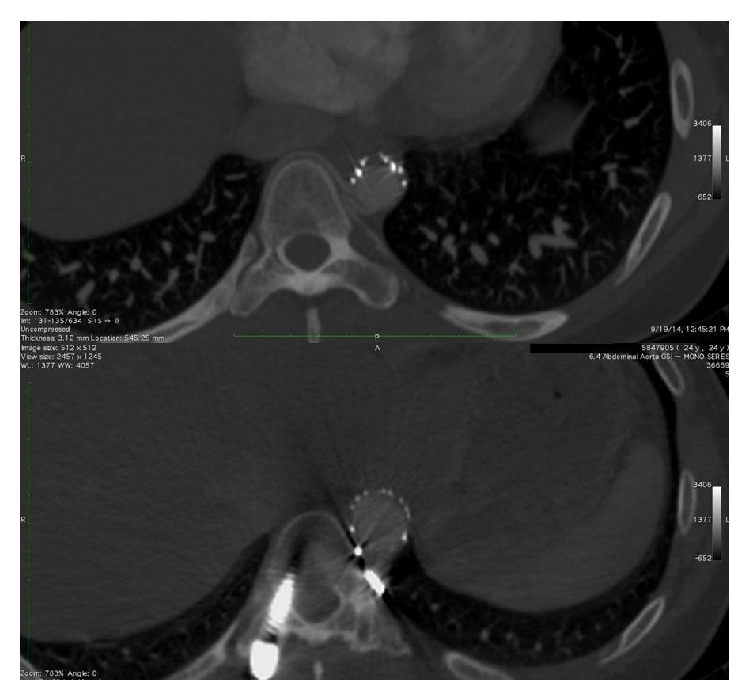
Two-year postoperative CT reveals the patency of the intercostal vessels and the restoration of the thoracic aorta.

The aim of the procedure was the safe removal of the screw. For this reason, a two-step approach was planned. The first step included the embolization of the left T10 intercostal artery (Simmons 4 Fr, Echelon 10, Axium coil) in order to avoid life-threatening bleeding during the removal of the pedicle screw (Figure 2). Having secured the safety of the intercostal artery, the team proceeded to the endovascular aortic repair, six weeks later. A short straight thoracic endovascular covered stent graft (Cook, 24 mm × 45 mm) was introduced via the right transfemoral approach into the thoracic aorta, at the level between T9 and T11, protecting the patency of the Adamkiewicz artery and the posterior radiculomedullary arteries at the above mentioned levels. An angiography was intraoperatively performed through the left femoral artery, where the stent graft was found accurately centered on the site of invasion of the screw (Figure 2). Simultaneously, through the left femoral artery, a super compliant balloon catheter was inserted in order to fix the stent graft on the aortic wall and to prevent a possible Type I endoleak. The patient was then turned to the right decubitus and spine surgeons removed the screw, which was responsible for the aortic injury. A completion angiogram via the left angiographic catheter demonstrated the correct position of the stent graft and the patency of the aortic vessels. The catheters were removed, the femoral arteriotomies and punctures were successfully repaired and the incisions in the groins were closed. The patient had an uneventful postoperative period, without any neurologic, systemic, and access site complications. A postoperative Dyna-CT scan revealed the correct position of the aortic endograft and the absence of hemorrhage around the aortic injury. The patient had a two-year CT follow-up and restoration of the patency of the aorta was revealed (Figure 3).

## 4. Discussion

Iatrogenic aortic injuries are well-recognized complications after spinal surgery. In most cases they are caused by screw misplacement and aortic perforation, which are attributed to an altered spatial relationship between the spine and the aorta in patients undergoing spinal fixation. Postoperative CT scanning studies reported a 4% to 25% incidence of pedicle screw misplacement following instrumentations for scoliosis [4, 5].

This is the only reported case in literature, presenting a significant interval between iatrogenic aortic injury and diagnosis without pseudoaneurysm formation. However, the contact between the pulsating aorta and the hardware could result in the perforation of the aorta, a complication that can be life threatening. This possible evolution explains the necessity of the removal of the hardware as soon as an aortic injury is diagnosed, even in case of asymptomatic patient [6, 7].

According to literature, endovascular treatment is the most common therapy for delayed iatrogenic thoracic injuries [8, 9]. This method was also selected in our case. Our primary goal was to protect the patient from hemorrhage, caused either by the aorta perforation or by the dislodgement of the thoracic intercostal artery, during the screw removal. For this reason, the T10 intercostal artery was embolized, reducing the risk of back flow leak. Several weeks later the patient underwent the main surgery. Moreover, the aorta was protected from possible hypertensive events with the use of a short endograft as indicated in literature [10]. Another important issue examined during the surgery was the preservation of the normal spinal cord blood flow through the Adamkiewicz artery and the radiculomedullary arteries, by placing the appropriate stent graft.

The weakness of this case was the long interval between the two steps. In similar cases [3, 6, 11] in reported literature, the second step is performed after 7 days (maximum). Nevertheless, no one reported injury and consequently embolization of the intercostal artery that requires several days of follow-up before proceeding to the next step. In addition, after the first step, the patient needed psychological support and she did not agree to continue to the next step, until 6 weeks later. However, she was hemodynamically stable and under close surveillance.

Based on our experience, the use of a two-step approach including embolization of the involved branch of the main injured vessel is a prerequisite for a safe main surgery. The preservation of the Adamkiewicz artery and the radiculomedullary artery, using the appropriate length of the stent graft, is the cornerstone of the endovascular treatment.
